# Inhibition of bone morphogenetic protein signaling prevents tau pathology

**DOI:** 10.1002/alz.090468

**Published:** 2025-01-03

**Authors:** Amira Affaneh, Anne K Linden, Elif Tunc‐Ozcan, Yung‐Hsu Tsai, Chian‐Yu Peng, John A Kessler

**Affiliations:** ^1^ Northwestern University, Chicago, IL USA

## Abstract

**Background:**

Aging is the most significant risk factor for neurodegenerative tauopathies, including Alzheimer’s disease (AD), frontotemporal dementia (FTD), progressive supranuclear palsy (PSP), and others. However, no specific age‐related molecular change in the brain has been identified that leads to disease onset and progression. We have found age‐related increases in bone morphogenic protein (BMP) signaling in both human and mouse brains. Inhibition of BMP signaling in mice improves cognitive performance and age‐related molecular changes in the brain. Therefore, we hypothesized a potential link between the age‐related increase in BMP signaling and the onset and progression of neurodegenerative pathology.

**Method:**

Neurons differentiated from iPSC‐derived (iNs) from AD individuals were used to investigate the effects of BMP signaling on tau phosphorylation and release and the mechanisms underlying these effects. Using mouse models, we overexpressed of BMP4 in wild‐type (WT) mice and inhibition of BMP signaling in the P301S (PS19) tauopathy mouse model were used to understand the effects of BMP signaling on cognition and pathology.

**Result:**

BMP signaling increased levels of phospho‐tau (p‐tau) expression and release in healthy control and AD iNs, and treatment with the BMP inhibitor, noggin, reduced p‐tau levels. The effects of BMP signaling on tau phosphorylation were mediated by increased non‐canonical p38 mitogen‐activated protein kinase signaling. APOE4 iNs have increased cellular and secreted p‐tau compared to isogenic cells that express APOE3, and the APOE4‐associated increase in p‐tau is further exacerbated by BMP4 treatment. However, noggin treatment reduced p‐tau in APOE4 cells to levels comparable to those in isogenic APOE3 cells. BMP4 overexpression in the brain increased p‐tau in WT mice *in vivo*. Remarkably, noggin overexpression prevented tau hyperphosphorylation, neuropathological changes, and behavioral abnormalities in the widely studied PS19 tauopathy mouse model.

**Conclusion:**

Evidence from mouse models and iPSC‐derived human neurons strongly supports the hypothesis that increased BMP signaling is part of aging‐related changes predisposing to tauopathies. Further, interactions between BMP signaling and genetic factors such as APOE4 act synergistically to increase tau phosphorylation and release. These observations suggest therapeutic interventions that reduce BMP signaling in the aging brain could potentially slow or prevent disease development.

